# Stabilization of Polymeric Nanofibers Layers for Use as Real-Time and In-Flow Photonic Sensors

**DOI:** 10.3390/s19183847

**Published:** 2019-09-06

**Authors:** Salvador Ponce-Alcántara, Paula Martínez-Pérez, Ana Pérez-Márquez, Jon Maudes, Nieves Murillo, Jaime García-Rupérez

**Affiliations:** 1 Nanophotonics Technology Center (NTC), Universitat Politècnica de València, 46022 Valencia, Spain (S.P.-A.) (P.M.-P.); 2 TECNALIA Research & Innovation, Mikeletegi Pasealekua, 2, 20009 Donostia-San Sebastián, Spain (A.P.-M.) (J.M.) (N.M.)

**Keywords:** nanofibers layers, polymers, optical sensor, thermal treatment, refractive index

## Abstract

In order to increase the sensitivity of a sensor, the relationship between its volume and the surface available to be functionalized is of great importance. Accordingly, porous materials are becoming very relevant, because they have a notable surface-to-volume ratio. Moreover, they offer the possibility to infiltrate the target substances on them. Among other porous structures, polymeric nanofibers (NFs) layers fabricated by electrospinning have emerged as a very promising alternative to low-cost and easy-to-produce high-performance photonic sensors. However, experimental results show a spectrum drift when performing sensing measurements in real-time. That drift is responsible for a significant error when trying to determine the refractive index variation for a target solution, and, because of that, for the detection of the presence of certain analytes. In order to avoid that problem, different chemical and thermal treatments were studied. The best results were obtained for thermal steps at 190 °C during times between 3 and 5 h. As a result, spectrum drifts lower than 5 pm/min and sensitivities of 518 nm/refractive index unit (RIU) in the visible range of the spectrum were achieved in different electrospun NFs sensors.

## 1. Introduction

In the race to provide high-sensitivity sensors with a reduced response time and price, photonics is becoming a notable option to be taken into account. In this respect, the use of optical sensors provides important advantages compared to other technologies, including a small size, a light weight, high sensitivity, a shorter time-to-result, label-free detection, a requirement for very low volumes of sample and reagents, resistance to hazardous and harsh environments, and immunity to electromagnetic interference [1]. Because of that, photonic sensors have been designed and developed for the detection of substances/analytes in many laboratories and industrial environments, such as clinical diagnosis, pharmaceutical and drug analysis, chemical and biological warfare agent detection, pollution monitoring, and food control [2,3,4,5,6]. In some photonic sensors, we can distinguish between the guiding and the sensing (external) media. In these sensors, the sensing measurement is based on the interaction of the evanescent part of a guided mode with the sensing medium. For this reason, a change in the sensing medium leads to a variation of the evanescent field properties, which are translated into a change of the effective refractive index [7,8]. Nevertheless, only the lower-intensity evanescent field propagating outside the optical structure is used for sensing purposes, which notably limits the sensitivity of this kind of sensor. In order to overcome this restriction, and to use the higher-intensity optical field located inside the structure for sensing purposes, porous materials are being extensively studied for the development of optical sensing structures [9,10,11]. Porous materials provide extremely high surface areas within small volumes, where a huge amount of biorecognition elements can be immobilized, thus implying an enhancement of the sensitivity of the optical sensor.

However, the active surface area can be restricted due to insufficient liquid diffusion inside the porous structure because of small pore diameters and/or the impossibility to evacuate the air that was initially filling the pores [11]. In order to overcome this limitation, polymeric nanofibers (NFs) layers fabricated by electrospinning are emerging as a very promising alternative to low-cost and easy-to-produce high-performance optical sensors. Polymeric NFs layers exhibit many interesting properties that make them ideal candidates to build optical sensing structures, such as a high surface-to-volume ratio, a high porosity, an adjustable pore size, a sponge-like configuration for a better sample infiltration, a low production cost, and the possibility to be manufactured over large areas [12,13]. High sensitivities (close to 1060 nm/RIU (refractive index unit) in the near-infrared range of the spectrum) have been previously demonstrated by our group in a static test consisting of the deposition of a drop of acetone over the porous NFs sample [14]. However, a spectrum drift has been observed when trying to perform real-time sensing measurements. In this work, we studied different chemical and thermal procedures for the structural stabilization of the NFs layer in order to overcome this problem. As a result, almost zero spectrum drift (<5 pm/min) was achieved during real-time and in-flow sensing tests. Sensitivities of 518 nm/RIU were achieved when using different stabilized samples, thus confirming the possibilities of this new type of porous photonic structure to perform sensing measurements.

## 2. Optical Sensors Based on Electrospun Nanofibers Layers

**Figure 1:**
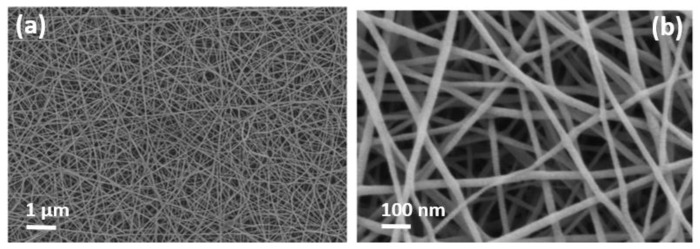
FESEM images of a typical nanofibers (NFs) layer used in this study. Two different magnifications have been used: (**a**) 10K (scale bar 1 μm); and (**b**) 100K (scale bar 100 nm).

Porous optical sensors were built from polymeric NFs layers fabricated by electrospinning. These layers have been deposited over a polished silicon wafer, and act like a Fabry–Pérot (FP) interferometer. NFs layers with average diameters between 22 and 40 nm and high porosities were achieved with an electrospinning solution, realized with 6 wt% polyamide 6 (PA6) and 5 wt% pyridine salt. Further details about the fabrication process of these samples can be found in [14]. Then, a thin (~3 nm) gold layer was deposited on top of the NFs layers in order to increase the amplitude of the FP fringes when measuring in aqueous medium. Their microstructural characterization was performed by a Field Emission Scanning Electron Microscope (FESEM) (Zeiss Ultra 55) as shown in Figure 1.

**Figure 2:**
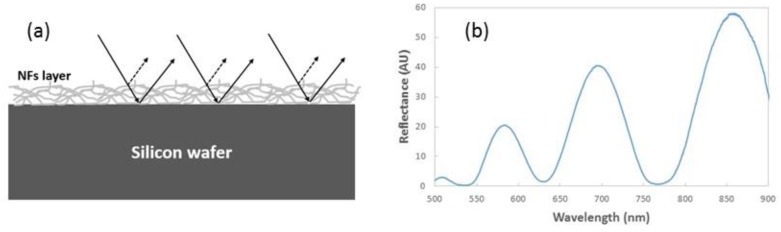
(**a**) Schematic diagram of a Fabry–Pérot (FP) NFs interferometer. (**b**) Reflectance spectrum of a polyamide 6 (PA6) NFs layer with a thin (~3 nm) gold layer deposited on top.

The FP interferometer was selected from all possible configurations due to the good relationship between fabrication complexity, cost, and sensitivity [15]. According to Figure 2a, this type of optical sensing structure has a reflectance spectrum that is characterized by the presence of interference fringes as a result of the constructive and destructive interferences produced by the light reflected at the interfaces between the FP layer and the upper/lower substrates. Figure 2b shows the reflectance spectrum in the visible region of a typical FP NFs layer fabricated using the above-described process. The spectrum was measured using a VIS-NIR spectrometer from Ocean Optics, model Flame T. The wavelength range for reflectance measurements was from 500 to 900 nm, with a resolution of 215 pm. Five consecutive spectra were measured every 2 ms, and then averaged to provide the final result. The Transfer Matrix Method (TMM) [16] was used to estimate the effective refractive index and the thickness of the porous samples from those reflectance measurements. Average values in the ranges 1.18–1.25 and 1190–1360 nm were obtained for these two parameters in different NFs layers. Thicknesses have been confirmed with experimental measurements carried out with a Veeco Dektak 150 surface profilometer.

## 3. Experimental Setup and Initial Tests

**Figure 3:**
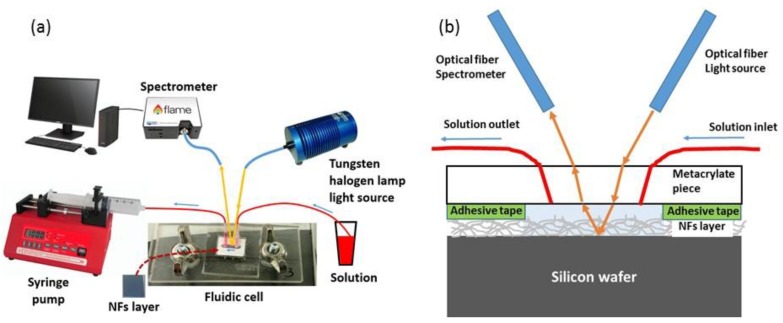
(**a**) Scheme of the opto-fluidic setup used to carry out the sensing experiments in real-time. (**b**) Sketch of the sensing region.

Figure 3 shows a scheme of the opto-fluidic setup used to perform the sensing experiments, using the FP NFs sensing layers in real-time. The NFs layer to be characterized was placed between two transparent methacrylate pieces. In order to flow different solutions over the sensor, a 1 cm^2^ adhesive tape with a 0.3 × 2.0 × 7.0 mm (height × width × length) channel defined on its center was fixed to the upper methacrylate piece. Fluids were introduced to and extracted from the channel through the fluidic tubes connected to the solution to be analyzed in one side, and to a syringe pump on the other side. The pump was configured in withdraw mode, in order to generate the vacuum necessary to suck the solutions from the vial located at the end of the microfluidic system. The pump was set to a constant flow rate of 20 μL/min during the whole experiment. Two different VIS-NIR optical fibers with a core of 400 μm were used to transmit the light from the light source to the sensing channel, and the reflected light to the spectrometer, respectively. These optical fibers were placed in the arms of a homemade goniometer. It allows for the use of different collimating lenses in each optical fiber, improving the spatial resolution of our setup. The angle selected for each arm was 15° with respect to the perpendicular axis of the NFs layer. The VIS-NIR spectrometer from Ocean Optics indicated in the previous section was used to collect the sample’s reflectance in real time.

**Figure 4:**
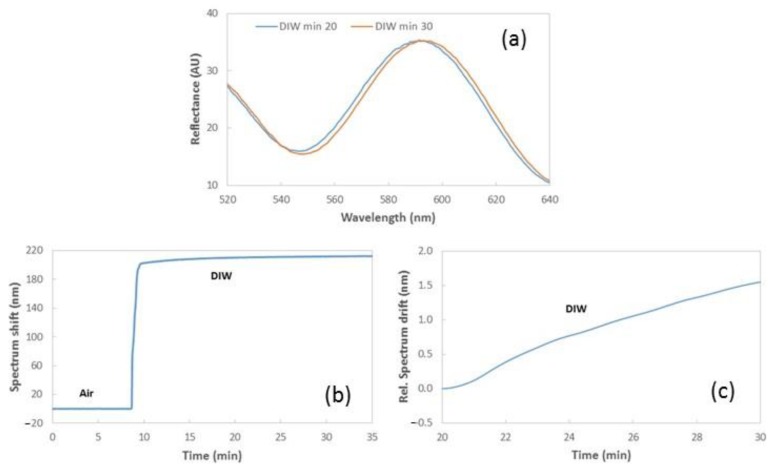
(**a**) Reflectance spectrum of a reference PA6 NFs layer when flowing deionized water (DIW) at two different moments of our sensing test. (**b**) Temporal evolution of the spectrum shift measured for the NFs layer in real-time, when changing from air to DIW flow. (**c**) Relative spectrum drift when flowing DIW between minutes 20 and 30. The peak considered to track the sensor response in (**b**,**c**) is located around 590 nm.

The reflectance spectrum of the FP NFs structure will shift to longer wavelengths depending on the refractive index increase of the fluids filling the porous structure. However, it was found that a spectrum shift is produced during the continuous flow of a given fluid, when no refractive index variations are produced in the sensor. As can be observed in Figure 4, the spectrum measured while flowing deionized water (DIW) over the FP NFs layer suffers a significant shift (~1.5 nm) after a 10-min period. As can also be observed, this shift is linear with time, indicating a drift in the range of 150 pm/min.

**Figure 5:**
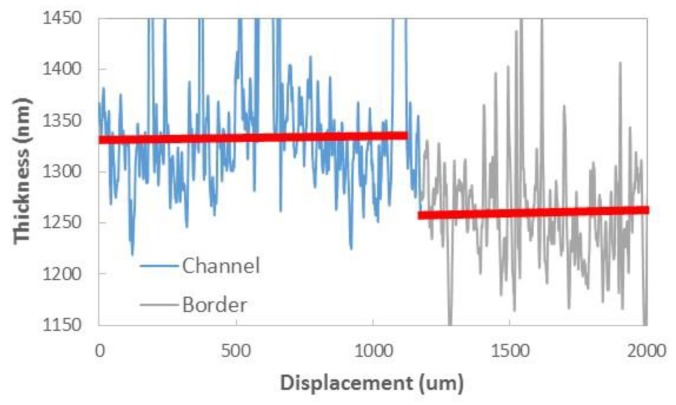
Profilometer measurement of the sensing channel and its border, in a NFs layer in which DIW has flowed for 6 h. Median values of each region are presented with solid lines.

As the refractive index of the medium filling the structure did not change, the observed drift should come from structural changes in the NFs layer itself. In this respect, Figure 5 shows a profilometer measurement of an NFs layer after a test flowing DIW for 6 h. It can be clearly observed that the thickness of the NFs layer has increased in the channel region with respect to the region where DIW did not flow.

Therefore, in order to improve the structural stability of NFs layers (with a minimum variation in their porosity), and thus to reduce that drift, different chemical and thermal processes were carried out. At least two NFs layers were used in each process. The most relevant results are displayed below.

## 4. Enhancement of the Structural Properties of NFs Layers with Chemical Treatments

### 4.1. Solvent Vapor Treatment

Solvent vapor treatment consists of exposing the nonwoven net of NFs to the vapor of the solvent used for its deposition, with the objective of welding the contact points of the NFs. The vapor melts these areas and allows them to fuse, which ultimately provides an improvement in the structural stability without varying the NFs layer’s structure [17].

In order to apply the solvent vapor treatment to the PA6 NFs, 25 μL of pure acetic acid was placed in a vial, which was covered by placing the sample face down. It was kept at room temperature for 15, 30, 45, or 60 min. Afterwards, samples were removed from the top of the vial and kept at room temperature in order to remove possible traces of acetic acid vapor on them. No important variations in the reflectance or in the layer thickness were found in any case. The average NFs diameter was obtained from FESEM images, observing that the diameter was slightly reduced from around 33 nm for the reference samples to around 31 nm.

Samples exposed for 60 min were used to perform sensing measurements in real-time. An average spectrum drift close to 120 pm/min was obtained during the test measurement with flowing DIW. This value was only slightly lower than the one obtained for the reference samples, which implies that some NFs were being fixed between them, improving the structural stability. However, that improvement was not enough to avoid the spectrum drift during a sensing measurement.

### 4.2. Hydrolysis and Chemical Crosslinking of Nanofibers

**Figure 6:**
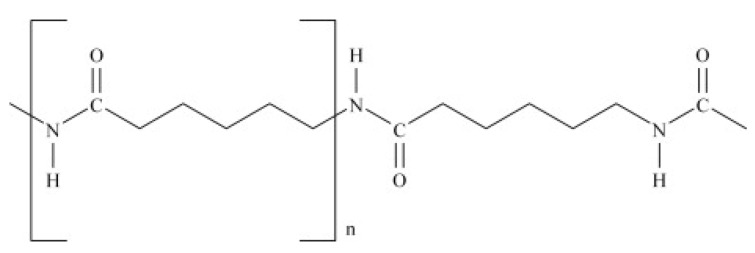
The chemical structure of PA6.

The chemical formula of PA6 is (C_6_H_11_NO)_n_. Its structure is shown in Figure 6, with the repeating unit in the brackets [18].

PA6 is built from monomeric units bound by peptide bonds that can be hydrolyzed. This reaction results in the exposure of carboxyl and amino groups, which are able to react and form new peptide bonds if they are correctly activated [19]. This strategy is normally used in order to bind proteins or any other biomolecule to the surface of PA6 supports [20]. However, in our approach, we aimed at crosslinking the NFs at the contact points by crosslinking carboxyl and amine groups exposed at such regions after hydrolyzing the NFs. For this aim, we have employed glutaraldehyde as a crosslinking agent.

**Figure 7:**
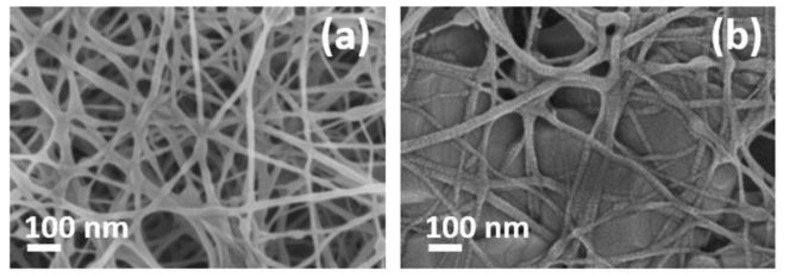
FESEM images of a PA6 NFs layer (**a**) before and (**b**) after the hydrolysis treatment.

Hydrolysis ought to be well-controlled in order to hydrolyze only some peptide bonds of the NFs and avoid a loss of their stability. For this reason, we exposed the NFs to hydrochloric acid (HCl) vapor for 15 min. Immediately after, we rinsed them with deionized water, and dried them with a soft airflow. Then, we immersed the NFs in 2.5% glutaraldehyde and 1x phosphate buffered saline (PBS) for 30 min and, after this time, we rinsed the samples with 1x PBS and dried them with a soft airflow. As a result, the layer thickness was reduced by around 35%, and the average diameter increased from around 33 nm to around 39 nm. According to Figure 7, it seems that the NFs layer was partially dissolved, giving a reduction in its porosity.

Different sensing measurements were done in real-time using these NFs samples. The spectrum drift was reduced to around 80 pm/min during the test measurement with flowing DIW, which is still high for our purpose. Moreover, due to the reduction in the layer’s thickness and porosity, this method was discarded to improve the structural stability of photonic sensors based on NFs layers.

As a summary of both chemical treatments studied in this section, Table 1 shows the most important achieved results.

## 5. Enhancement of the Structural Properties of NFs Layers with Thermal Processes

**Figure 8:**
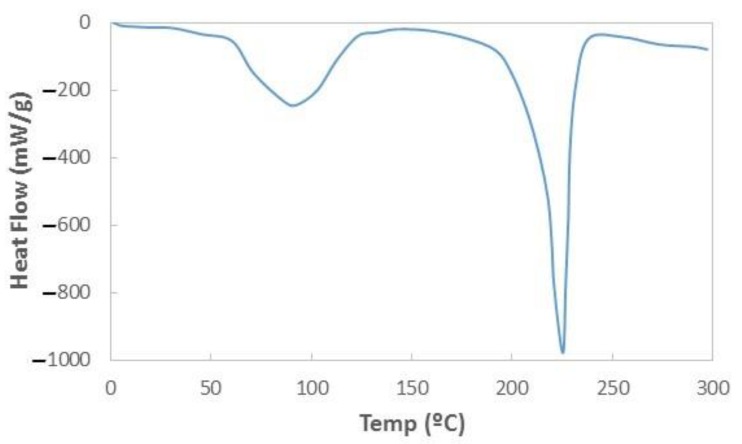
Differential scanning calorimetry (DSC) thermogram of a reference PA6 NFs layer used in our sensing tests.

Different thermal treatments were done in order to improve the structural stability of PA6 NFs layers. As a starting point, a differential scanning calorimetry (DSC) analysis was done in order to know the melting temperature of our PA6 NFs layer. DSC is a thermal analysis apparatus that measures how the physical properties of a sample change, along with temperature against time [21]. It was performed to demonstrate thermal effects of the materials whose melting behavior was analyzed systematically. Figure 8 shows the result achieved in one of our PA6 NFs layers. The DSC plot shows a decrease in heat flows at a temperature close to 90 °C, which is associated with an imperfect or a very small crystalline nucleus with a low fusion energy. Moreover, there is a notable endothermic peak at temperatures close to 225 °C due to the melting of the NFs layer.

Our objective was to melt the NFs layer lightly, allowing NFs to be fixed between them. Because of that, and taking into account the DSC results, five different furnace treatments were done with temperatures between 180 °C and 200 °C (lower than the melting one), and times between one and five hours. A chamber furnace from Selecta Group, model 210, was used to this end. A pressure of ~500 gr/cm^2^ was applied on the samples, placing a glass slide to protect the NFs layer and the weight on them. Table 2 shows a summary of the most important achieved results.

**Figure 9:**
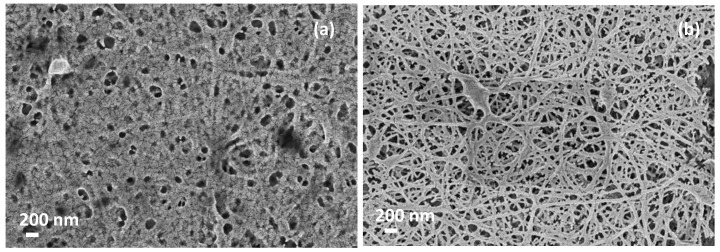
FESEM images of NFs layers after a heat treatment at (**a**) 200 °C for five hours and (**b**) 190 °C for three hours.

In the first heat treatment carried out, the temperature and time selected were 200 °C and five hours, respectively. There was no spectrum shift during the sensing measurement in real-time. An FESEM image (Figure 9a) confirms that the NFs layer has been partially melted, and, because of that, the liquid cannot be introduced into the NFs structure. The best results were obtained for thermal treatments at 190 °C during three and five hours. Figure 9b shows an FESEM image of a sample heated for three hours, where it can be observed that NFs are starting to be melted. Hence, a temperature of 190 °C for three hours was used for welding the NFs layers.

**Figure 10:**
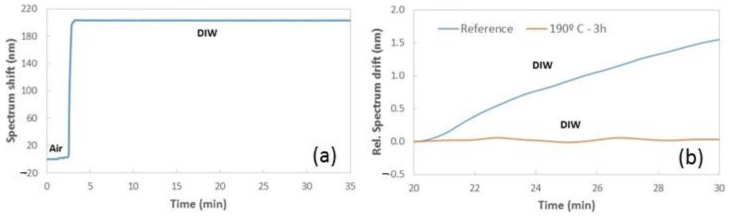
(**a**) Temporal evolution of the spectrum shift when flowing DIW in real-time for a PA6 NFs layer, after a heat treatment at 190 °C for three hours. (**b**) Comparison of the relative spectrum drift when flowing DIW, between minutes 20 and 30, for the reference sample and the one heated at 190 °C for three hours.

In this respect, Figure 10a shows the spectrum shift when flowing DIW in real-time and Figure 10b compares the drift obtained in this case with the one of the reference NFs layer, without any thermal treatment. As has been indicated in Table 2, the spectrum drift was reduced to almost zero after applying this thermal treatment.

## 6. Sensing Tests

Taking the previous results into account, three NFs layers with average thicknesses between 1190 and 1360 nm and effective refractive indices between 1.18 and 1.25 were prepared with the abovementioned thermal process at 190 °C for 3 h. Afterwards, real-time sensing measurements were carried out. Different concentrations of ethanol between 2.5% and 0.1% were selected. Their refractive indices were obtained from [22].

**Figure 11:**
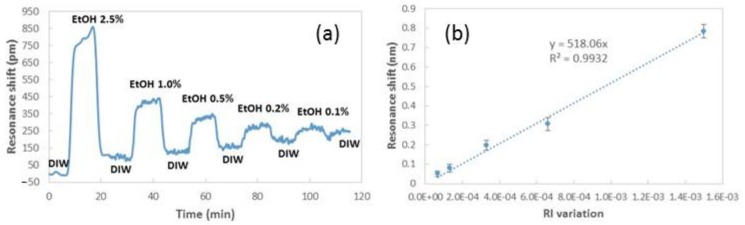
(**a**) Temporal evolution of the spectrum shift measured for a PA6 NFs sensing layer in real-time. (**b**) Sensitivity curve of three different sensing tests realized with different NFs layers.

During the real-time sensing measurement, the spectra were captured with a period of four seconds for the initial cycle with DIW and 2.5% EtOH, and with a period of one second in the following cycles. Figure 11 shows the temporal evolution of the spectrum shift of one of the NFs layers, as well as the sensitivity curve determined from the spectral shifts that were experimentally observed on three different sensing tests with three different NFs layers.

The average sensitivity achieved was close to 518 nm/RIU in the visible range of the spectrum. Moreover, sensing measurements were carried out with a spectrum drift lower than 5 pm/min.

## 7. Conclusions

In this work, we have demonstrated that a PA6 NFs layer is an interesting alternative as a porous photonic sensor, due to its low cost, high porosity, the possibility to be manufactured in large areas, its sponge-like structure for better sample infiltration, its high sensitivity, and the option to be used in the visible range of the spectrum, which provides the possibility to use low-cost characterization tools. However, the performance of this type of optical structure for real-time and in-flow measurements is limited by the significant spectral drift experienced during the realization of these experiments (around 150 pm/min). This spectral drift is provoked by the limited structural stability of the NFs net created during the electrospinning process.

In order to solve this structural instability issue, we have tested different conditioning processes. When using chemically based approaches, such as those based on the treatment with solvent vapor or the hydrolysis and chemical crosslinking, some reduction of the spectral drift was achieved, but it was still noticeable. On the other hand, the application of a treatment based on increasing the temperature to a value close to the melting point of the PA6 NFs, while applying pressure to the layer, has allowed us to reduce the spectral drift to almost zero by properly selecting the treatment conditions.

We were able to perform real-time and in-flow refractive index sensing experiments using several NFs layers that were treated using the selected conditioning process, with very repeatable results. From the obtained results, we have determined an experimental sensitivity of 518 nm/RIU for the NFs sensors that were developed in this work.

## Figures and Tables

**Table 1:** Crosslinking conditions and morphological evaluation of NFs layers after the chemical treatments.

NFs Layers Group	Crosslinking Conditions	Exposure Time	Average NFs Diameter (nm)	NFs Layer Thickness Variation	Spectrum Drift (pm/min)
REF	—	—	~33	—	~150
A	Acetic Acid vapor	1 h	~31	~0%	~120
B	HCl + glutaraldehyde	15′ HCl + 30′ 2.5% glutaraldehyde	~39	~−35%	~80

**Table 2:** Crosslinking conditions and morphological evaluation of NFs layers after the thermal treatments.

NFs Layers Group	Crosslinking Conditions	Exposure Time	Average NFs Diameter (nm)	NFs Layer Thickness Variation	Spectrum Drift (pm/min)
REF	—	—	~33	—	~150
C	200 °C	5 h	Melted layer	~−45%	Melted layer
D	180 °C	5 h	~36	~−15%	~30
E	190 °C	5 h	~43	~−25%	~5
F	190 °C	3 h	~41	~−20%	~8
G	190 °C	1 h	~38	~−15%	~20
